# Phase 1/2 study of epacadostat in combination with ipilimumab in patients with unresectable or metastatic melanoma

**DOI:** 10.1186/s40425-019-0562-8

**Published:** 2019-03-20

**Authors:** Geoffrey T. Gibney, Omid Hamid, Jose Lutzky, Anthony J. Olszanski, Tara C. Mitchell, Thomas F. Gajewski, Bartosz Chmielowski, Brent A. Hanks, Yufan Zhao, Robert C. Newton, Janet Maleski, Lance Leopold, Jeffrey S. Weber

**Affiliations:** 1 Donald A. Adam Melanoma and Skin Cancer Research Center of Excellence, Moffitt Cancer Center, 12902 Magnolia Drive, Tampa, FL USA; 2 Melanoma and Skin Cancers Center, The Angeles Clinic and Research Institute, 11818 Wilshire Blvd, Suite #200, Los Angeles, CA USA; 3 Division of Hematology & Oncology, Mount Sinai Medical Center, 4306 Alton Rd, Miami Beach, FL USA; 4 Department of Hematology/Oncology, Fox Chase Cancer Center, 333 Cottman Ave, Philadelphia, PA USA; 5 Division of Hematology Oncology, Abramson Cancer Center, Hospital of the University of Pennsylvania, 3400 Civic Center Blvd, Philadelphia, PA USA; 6 Department of Hematology/Oncology, University of Chicago, 5841 S Maryland Ave, MC2115, Chicago, IL USA; 7 Jonsson Comprehensive Medical Center, University of California, Los Angeles, 10945 Le Conte Ave #2339, Los Angeles, CA USA; 8 Department of Medicine, Duke University Medical Center, 20 Duke Medicine Cir, Durham, NC USA; 9 Incyte Corporation, 1801 Augustine Cutoff, Wilmington, DE USA; 10 Present Address: Laura and Isaac Perlmutter Cancer Center, NYU Langone Medical Center, New York, NY USA; 11 Present Address: Lombardi Comprehensive Cancer Center, Medstar-Georgetown University Hospital, 3800 Reservoir Road NW, Podium A, Washington, DC 20007 USA

**Keywords:** Epacadostat, IDO1, Immune checkpoint inhibition, Ipilimumab, Melanoma

## Abstract

**Background:**

Epacadostat is a potent inhibitor of the immunosuppressive indoleamine 2,3-dioxygenase 1 (IDO1) enzyme. We present phase 1 results from a phase 1/2 clinical study of epacadostat in combination with ipilimumab, an anti-cytotoxic T-lymphocyte-associated protein 4 antibody, in advanced melanoma (NCT01604889).

**Methods:**

Only the phase 1, open-label portion of the study was conducted, per the sponsor’s decision to terminate the study early based on the changing melanoma treatment landscape favoring exploration of programmed cell death protein 1 (PD-1)/PD-ligand 1 inhibitor-based combination strategies. Such decision was not related to the safety of epacadostat plus ipilimumab. Patients received oral epacadostat (25, 50, 100, or 300 mg twice daily [BID]; 75 mg daily [50 mg am, 25 mg pm]; or 50 mg BID intermittent [2 weeks on/1 week off]) plus intravenous ipilimumab 3 mg/kg every 3 weeks.

**Results:**

Fifty patients received ≥1 dose of epacadostat. As of January 20, 2017, 2 patients completed treatment and 48 discontinued, primarily because of adverse events (AEs) and disease progression (*n* = 20 each). Dose-limiting toxicities occurred in 11 patients (*n* = 1 each with epacadostat 25 mg BID, 50 mg BID intermittent, 75 mg daily; *n* = 4 each with epacadostat 50 mg BID, 300 mg BID). The most common immune-related treatment-emergent AEs included rash (50%), alanine aminotransferase elevation (28%), pruritus (28%), aspartate aminotransferase elevation (24%), and hypothyroidism (10%). Among immunotherapy-naive patients (*n* = 39), the objective response rate was 26% by immune-related response criteria and 23% by Response Evaluation Criteria in Solid Tumors version 1.1. No objective response was seen in the 11 patients who received prior immunotherapy. Epacadostat exposure was dose proportional, with clinically significant IDO1 inhibition at doses ≥25 mg BID.

**Conclusions:**

When combined with ipilimumab, epacadostat ≤50 mg BID demonstrated clinical and pharmacologic activity and was generally well tolerated in patients with advanced melanoma.

**Trial registration:**

ClinicalTrials.gov identifier, NCT01604889. Registration date, May 9, 2012, retrospectively registered.

**Electronic supplementary material:**

The online version of this article (10.1186/s40425-019-0562-8) contains supplementary material, which is available to authorized users.

## Introduction

Cancer progression and metastasis can result from tumor cells evading immunosurveillance [1, 2]. The development of immunotherapy strategies that harness the immune system to counteract mechanisms exploited by tumors to evade immunity have the potential to improve cancer outcomes. Immune checkpoint inhibitors, such as cytotoxic T-lymphocyte-associated protein 4 (CTLA-4), programmed cell death protein 1 (PD-1), and PD-ligand 1 (PD-L1) inhibitors, counter immune checkpoint–mediated suppression of effector T-cell activity [1]. The anti-CTLA-4 antibody ipilimumab was the first immune checkpoint inhibitor shown to improve overall survival (OS) in a randomized, controlled phase 3 trial of patients with metastatic melanoma [3], resulting in a 2011 approval from the US Food and Drug Administration (FDA) to treat patients with late-stage (metastatic) melanoma [4]. Although promising, the beneficial outcomes associated with ipilimumab monotherapy are limited to a fraction of patients treated (11% and 11%–19% overall response rates for previously treated and treatment-naive patients, respectively, in previous studies) [3, 5, 6]. A combination immunotherapy strategy targeting more than 1 mechanism of immune evasion may be a more viable approach for increasing the proportion of patients responding to therapy and for achieving deeper and more durable responses in patients with advanced disease.

Based on preclinical murine data demonstrating synergy between immune checkpoint inhibitors and indoleamine 2,3-dioxygenase 1 (IDO1) enzyme inhibitors [7], this phase 1/2 clinical trial (NCT01604889) was initiated to investigate ipilimumab in combination with epacadostat for the treatment of unresectable or metastatic melanoma. Epacadostat is a potent and highly selective oral inhibitor of IDO1 [8], an intracellular enzyme that catalyzes the first and rate-limiting step of tryptophan (trp) degradation in the kynurenine (kyn) pathway [2]. Depletion of cellular trp and increased levels of downstream metabolites, including kyn, may contribute to a local immunosuppressive environment in the tumor microenvironment (TME), including inhibition of T-cell activation as well as induction of T-cell apoptosis and regulatory T cell differentiation [2, 9–11]. Furthermore, upregulation of IDO1 expression has been observed within the TME [12–14] and associated with inferior clinical outcomes in several tumor types, including melanoma [15–17], making IDO1 an attractive target for immunotherapy across a broad range of tumor types. In a first-in-human phase 1 study in patients with advanced solid tumors, epacadostat was generally well tolerated at dose levels predicted to yield clinically meaningful suppression of IDO1 activity [18]. Taken together, findings from these studies provided the basis for evaluating epacadostat as part of novel combination immunotherapeutic regimens, including the epacadostat and ipilimumab combination explored in the present study.

During the conduct of this trial, the emergence and clinical success of PD-1 inhibitors led to a shift in the melanoma treatment landscape. These newer agents, pembrolizumab and nivolumab, demonstrated superior efficacy and improved safety profiles compared with ipilimumab [19–22]. Based on such evolving immunotherapy landscape, this study was terminated early, after a dose of epacadostat was identified for potential phase 2 testing in combination with ipilimumab.

## Methods

### Study design and treatment

This protocol was originally designed as a phase 1/2 study of patients with unresectable or metastatic melanoma (Additional file 1: Figure S1). Phase 1 was planned to include epacadostat dose-escalation and dose-expansion portions during which patients were to receive ipilimumab in combination with open-label epacadostat. Phase 2 was designed to be randomized, blinded, and placebo-controlled to evaluate ipilimumab in combination with epacadostat or placebo. The emerging success of PD-1 inhibitors in treating advanced melanoma led to the study sponsor’s decision to suspend further patient enrollment in phase 1 dose expansion; no patients were enrolled into phase 2. The phase 1 dose-escalation portion of the study described here was conducted in accordance with the provisions of the Declaration of Helsinki, as described in the International Council for Harmonisation Guidelines for Good Clinical Practice, and was approved by the institutional review board at each participating institution. All patients provided informed consent before initiation of treatment.

Based on the safety, pharmacokinetic, and pharmacodynamic findings of epacadostat monotherapy in the phase 1 first-in-human study [18], two initial dose schedules of epacadostat (300 mg and 600 mg twice daily [BID]) were planned for evaluation in combination with intravenous ipilimumab 3 mg/kg every 3 weeks (Q3W) in a 6 + 6 dose-escalation design. Epacadostat dosing was to have been escalated if fewer than 2 of the first 6 evaluable patients or (when an additional 6 evaluable patients were enrolled) fewer than 3 of 12 evaluable patients experienced a dose-limiting toxicity (DLT). Dose interruptions, reductions, and discontinuations for epacadostat and dose interruptions and discontinuations for ipilimumab were allowed if patients experienced protocol-defined toxicity. A few of the first patients who received an epacadostat starting dose of 300 mg BID began to experience clinically significant alanine aminotransferase (ALT) elevations, resulting in dose reductions to epacadostat 100 mg BID and a protocol amendment to lower the epacadostat starting dose to 100 mg BID, under which 1 patient was enrolled. When 5 of 7 patients who initially received epacadostat 300 mg BID in combination with ipilimumab developed clinically significant ALT elevations (including 2 patients who experienced dose-limiting ALT elevations after having their doses reduced to 100 mg BID; ALT elevations were reversible with corticosteroids upon treatment discontinuation), the investigators and sponsor stopped epacadostat treatment and further enrollment in the study, which was subsequently placed on clinical hold for 6 months by the FDA. The study then reopened after the protocol was amended to include additional pharmacovigilance monitoring and to evaluate epacadostat doses ≤50 mg BID (25 mg BID, 50 mg BID continuous, 50 mg BID intermittent [2 weeks on/1 week off], and 75 mg daily [50 mg am, 25 mg pm]) plus ipilimumab 3 mg/kg Q3W. Epacadostat doses ≥100 mg BID were not explored further in this study. After completing 12 weeks (four 21-day cycles) of the combination treatment, patients who received benefit could continue treatment with epacadostat monotherapy at their assigned dose (or a lower dose if needed based on safety findings) for up to 2 years.

### Study population

Men and women ≥18 years of age with unresectable or metastatic melanoma, a life expectancy >12 weeks, and an Eastern Cooperative Oncology Group performance status of 0 or 1 were eligible to participate. Patients must have been tested for the V600E activating *BRAF* mutation. Laboratory and medical history parameters were required to be within normal institutional ranges. Patients were treatment-naive or previously treated for unresectable or metastatic disease. Prior immune checkpoint inhibitor therapy (eg, anti−CTLA-4, anti–PD-1, anti–PD-L1 monoclonal antibody) was permitted for patients without associated protocol-defined grade 3/4 immune-related adverse events (irAEs).

Exclusion criteria included central nervous system metastasis (unless the patient had asymptomatic, clinically stable disease [defined as no increase in lesion size or number for ≥28 days following whole brain irradiation or relief of symptoms for ≥7 days following stereotactic radiosurgery or ≥28 days following surgical resection] not requiring steroids), unresolved grade >2 toxicities from anticancer therapy, grade 3/4 pneumonitis, autoimmune disease, and history of serotonin syndrome. Use of investigational study drugs within 28 days or 5 half-lives before screening (whichever was longer), other anticancer treatment within 21 days before receiving first study treatment dose (or 6 weeks for mitomycin C and nitrosoureas), and immunologically based treatments (including chronic systemic steroid use at doses ≥7.5 mg/day prednisone equivalent, excluding inhaled or topical steroids) were not permitted. Additional exclusion criteria added during the course of the study included elevated levels of liver chemistries, extensive liver metastases, excessive alcohol intake, excessive chronic acetaminophen use (ie, >2 g/day) at screening, and history of hepatitis or positive serology for hepatitis B or C.

### Study assessments

The primary objectives were to evaluate the safety, tolerability, and DLTs associated with epacadostat plus ipilimumab. Safety and tolerability assessments included adverse event (AE) monitoring, comprehensive and targeted physical examination, vital signs, 12-lead electrocardiogram, assessment of serotonin syndrome symptoms [23], and clinical laboratory tests. Adverse events were assessed according to Common Terminology Criteria for Adverse Events version 4.03 on Days 1 and 10 of Cycle 1, Day 1 of subsequent treatment cycles, at end of treatment, and 1 and 2 months after the last treatment dose during follow-up. Dose-limiting toxicities were defined as the occurrence of any protocol-specified toxicity occurring during the first 8 weeks of treatment. Such toxicity could include grade 4 thrombocytopenia or neutropenia lasting >7 days; grade 4 nonhematologic toxicity; grade 3/4 aspartate aminotransferase (AST), ALT, or total bilirubin elevation; other nonhematologic grade 3 toxicity (excluding nausea/vomiting controlled by medical intervention within 72 h); documented infection (with or without fever) lasting ≥7 days; or grade ≥2 episcleritis, uveitis, or iritis. Immune-related AEs in this study included any previously observed with ipilimumab therapy [4] as well as any AEs considered related to the mechanism of action of epacadostat, ipilimumab, or other immune checkpoint inhibitors to capture any other autoimmune phenomena.

Secondary and exploratory objectives were to evaluate the preliminary efficacy of epacadostat plus ipilimumab based on assessments of objective response rate (ORR), duration of response (DOR), progression-free survival (PFS), and OS. Tumors were assessed by computerized tomography or magnetic resonance imaging (same scanning modality used throughout) at baseline and at tumor assessment study visits occurring every 9 weeks (for treatment Cycles 1–6) and every 12 weeks (starting on treatment Cycle 7), until disease progression, initiation of new anticancer therapy, or death. Tumor response, DOR, and PFS were evaluated according to immune-related response criteria (irRC) [24] and Response Evaluation Criteria in Solid Tumors version 1.1 (RECIST v1.1) [25]. Per irRC, patients were permitted to continue in the study after the first appearance of progressive disease (PD) at the investigator’s discretion as long as the patient was not clinically deteriorating and PD was not yet confirmed. For both irRC and RECIST v1.1, patients’ overall response was evaluated as complete response (CR), partial response (PR), stable disease (SD), progressive disease (PD), or not evaluable (NE) at each postbaseline radiologic assessment based on changes in target lesions, nontarget lesions, and the appearance of new lesions. A patient was considered to have disease control if they responded (CR, PR) or had SD at least 56 days after treatment start date. Duration of response was the time from the first objective response to the first documented evidence of PD or death. Progression-free survival was defined as the time between the treatment start date and PD or death, whichever occurred earlier. Overall survival was defined as the number of days from the treatment start date to death. Patients were followed for survival every 3 months (up to a maximum of 2 years) after study drug discontinuation.

Plasma and whole blood samples were collected pre-dose and post-dose at protocol-defined time points for pharmacokinetic and pharmacodynamic analyses. Epacadostat pharmacokinetics were assessed by validated liquid chromatography with tandem mass spectrometry (LC-MS/MS) assay. Changes in plasma protein analytes were measured by an enzyme-linked immunosorbent assay or other relevant methods by Incyte Corporation (Wilmington, DE) or Incyte’s designee. Plasma levels of trp and kyn were evaluated using LC-MS/MS. Whole blood samples were stimulated with interferon-γ (100 ng/mL) and lipopolysaccharide (100 ng/mL) for 18 h to induce IDO1 expression; trp and kyn levels were subsequently measured by LC-MS/MS. For each patient, the relative IDO1 inhibition was calculated as the percentage reduction in kyn levels between pre-dose and post-dose samples.

Tumor biopsies were optional in phase 1 of this study. Therefore, although immunohistochemical staining of tumor biopsies to determine if the primary tumor or stromal cells expressed IDO1 was planned, an insufficient number of samples were collected to conduct a meaningful analysis.

### Statistical analyses

Final study results are reported based on a January 20, 2017, data cutoff. Safety and efficacy analyses included patients receiving ≥1 dose of epacadostat. The pharmacokinetic/pharmacodynamic–evaluable population included patients who received ≥1 dose of epacadostat and provided ≥1 post-dose plasma sample for analysis.

SAS® software, version 9.4 (SAS Institute Inc., Cary, NC) was used to generate all tables, graphs, and statistical analyses. Epacadostat pharmacokinetics was estimated by noncompartmental model analysis (Phoenix WinNonlin version 6.0 or later; Pharsight Corporation, Mountain View, CA). Descriptive statistics were used to present summaries of continuous and categorical variables. Survival data (OS, PFS) were analyzed using the nonparametric Kaplan-Meier method.

## Results

### Patient population

A total of 50 patients were enrolled in the study. The first 8 enrolled patients were treated with epacadostat 300 mg BID [*n* = 7] or 100 mg BID [*n* = 1] in combination with ipilimumab 3 mg/kg Q3W. Five of 7 patients treated with epacadostat 300 mg BID experienced clinically significant grade 3/4 ALT elevations (all reversible with corticosteroids upon treatment discontinuation), which led to an amendment in the study protocol to evaluate lower epacadostat doses. After the amendment, the study was restarted and patients were treated with epacadostat 25 mg BID (*n* = 8), 50 mg BID continuous (*n* = 18), 50 mg BID intermittent (2 weeks on/1 week off; *n* = 9), or 75 mg daily (50 mg am, 25 mg pm; *n* = 7) in combination with ipilimumab 3 mg/kg Q3W.

As of the January 20, 2017 data cutoff, 2 patients had completed ≥2 years of treatment, and 48 had discontinued treatment. The most frequent reasons for study treatment discontinuation were AEs (regardless of relation to study treatment) and disease progression (*n* = 20, 40% each). Additional reasons for discontinuation included withdrawal of consent (*n* = 4; 8%), sponsor decision (*n* = 3; 6% [patients in the discontinued 100 mg BID and 300 mg BID dose groups not experiencing a grade 3/4 ALT elevation]), and investigator decision (*n* = 1; 2%; Table 1). Twenty patients remained on study and completed the planned survival follow-up period.

**Table 1:** Patient Disposition

Disposition Status, n (%)	25 mg BID (*n* = 8)	50 mg BID Cont’ (*n* = 18)	50 mg BID Int’ (*n* = 9)	75 mg Total Daily (*n* = 7)	100 mg BID^a^ (*n* = 1)	300 mg BID^a^ (*n* = 7)	Total (*N* = 50)
Patients who completed study treatment	1 (12.5)	0	1 (11.1)	0	0	0	2 (4.0)
Patients who discontinued study treatment	7 (87.5)	18 (100.0)	8 (88.9)	7 (100.0)	1 (100.0)	7 (100.0)	48 (96.0)
Primary reason for discontinuation from treatment/early termination^b^							
Adverse event	4 (50.0)	8 (44.4)	3 (33.3)	0	0	5 (71.4)	20 (40.0)
Disease progression	3 (37.5)	8 (44.4)	4 (44.4)	5 (71.4)	0	0	20 (40.0)
Consent withdrawn	0	1 (5.6)	1 (11.1)	2 (28.6)	0	0	4 (8.0)
Sponsor decision	0	0	0	0	1 (100.0)	2 (28.6)	3 (6.0)
Investigator decision	0	1 (5.6)	0	0	0	0	1 (2.0)

*ALT* alanine aminotransferase, *AST* aspartate aminotransferase, *BID* twice daily, *cont’* continuous, *int’* intermittent

^a^ All patients who received epacadostat 100 mg BID and 300 mg BID discontinued treatment after 5 of these patients developed clinically significant ALT/AST elevations. These doses were not re-explored in this study after protocol amendment to evaluate lower epacadostat doses

^b^ No patients discontinued or terminated study treatment early because of death, lost to follow-up, noncompliance, patient decision, protocol deviation, or termination of the clinical study by the sponsor

Baseline demographic and disease characteristics for the 50 patients are shown in Table 2. Median (range) age was 63 (25–81) years, 64% of patients were men, and all were white. The majority of patients had stage IV melanoma (94%) and M1c disease (70%). The most common sites of metastases at baseline were the lymph nodes (56%), lung (52%), and liver (30%). Sixteen patients (32%) had *BRAF* mutations, and 12 (24%) had elevated lactate dehydrogenase levels. Before enrollment, 40% of patients had received radiation therapy for melanoma, and 46% had been treated with systemic therapy in the advanced or metastatic disease setting. Immune checkpoint inhibitors were the most common prior systemic treatments among these patients (*n* = 6; 12%). Thirty-nine patients were immunotherapy-naive for advanced or metastatic disease, and 11 had received prior immunotherapy (immune checkpoint inhibitor, interferon, IL-2, or other) for advanced or metastatic disease.

**Table 2:** Patient Demographics and Disease Characteristics at Baseline

	25 mg BID (*n* = 8)	50 mg BID Cont’ (*n* = 18)	50 mg BID Int’ (*n* = 9)	75 mg Total Daily (*n* = 7)	100 mg BID^a^ (*n* = 1)	300 mg BID^a^ (*n* = 7)	Total (*N* = 50)
Age							
Median (range), y	67 (34–81)	57 (25–78)	69 (49–77)	62 (35–81)	67 (67)	64 (47–81)	63 (25–81)
≤ 65 y, n (%)	3 (37.5)	14 (77.8)	2 (22.2)	4 (57.1)	0	5 (71.4)	28 (56.0)
White, n (%)	8 (100.0)	18 (100.0)	9 (100.0)	7 (100.0)	1 (100.0)	7 (100.0)	50 (100.0)
Men, n (%)	6 (75.0)	9 (50.0)	6 (66.7)	3 (42.9)	1 (100.0)	7 (100.0)	32 (64.0)
ECOG PS, n (%)							
0	5 (62.5)	16 (88.9)	6 (66.7)	6 (85.7)	1 (100.0)	4 (57.1)	38 (76.0)
1	3 (37.5)	2 (11.1)	3 (33.3)	1 (14.3)	0	3 (42.9)	12 (24.0)
Current staging, n (%)							
IIIB	1 (12.5)^b^	0	0	0	0	0	1 (2.0)
IIIC	0	0	2 (22.2)	0	0	0	2 (4.0)
IV	7 (87.5)	18 (100.0)	7 (77.8)	7 (100.0)	1 (100.0)	7 (100.0)	47 (94.0)
Current M classification, n (%)							
0	0	0	2 (22.2)	0	0	0	2 (4.0)
1a	1 (12.5)	3 (16.7)	0	2 (28.6)	0	2 (28.6)	8 (16.0)
1b	2 (25.0)	1 (5.6)	1 (11.1)	1 (14.3)	0	0	5 (10.0)
1c	5 (62.5)	14 (77.8)	6 (66.7)	4 (57.1)	1 (100.0)	5 (71.4)	35 (70.0)
Most common sites of metastases, n (%)							
Lymph nodes	3 (37.5)	12 (66.7)	4 (44.4)	3 (42.9)	0	6 (85.7)	28 (56.0)
Lung	4 (50.0)	13 (72.2)	4 (44.4)	3 (42.9)	1 (100.0)	1 (14.3)	26 (52.0)
Liver	2 (25.0)	6 (33.3)	3 (33.3)	3 (42.9)	0	1 (14.3)	15 (30.0)
Abdomen	0	3 (16.7)	3 (33.3)	0	0	3 (42.9)	9 (18.0)
Skin	1 (12.5)	4 (22.2)	1 (11.1)	1 (14.3)	0	2 (28.6)	9 (18.0)
Adrenals	1 (12.5)	3 (16.7)	2 (22.2)	0	1 (100.0)	0	7 (14.0)
CNS/brain	1 (12.5)	4 (22.2)	0	1 (14.3)	0	0	6 (12.0)
Chest wall	0	2 (11.1)	0	1 (14.3)	0	2 (28.6)	5 (10.0)
Breast	0	3 (16.7)	0	0	0	0	3 (6.0)
Peritoneum	0	0	1 (11.1)	1 (14.3)	0	0	2 (4.0)
Bone	0	0	0	0	0	1 (14.3)	1 (2.0)
Pancreas	0	1 (5.6)	0	0	0	0	1 (2.0)
Pleura	0	1 (5.6)	0	0	0	0	1 (2.0)
Other	5 (62.5)	6 (33.3)	1 (11.1)	2 (28.6)	0	3 (42.9)	17 (34.0)
*BRAF*-mutant positive, n (%)	2 (25.0)	8 (44.4)	2 (22.2)	1 (14.3)	1 (100.0)	2 (28.6)	16 (32.0)
Elevated LDH level, n (%)	2 (25.0)	3 (16.7)	3 (33.3)	2 (28.6)	0	2 (28.6)	12 (24.0)
Prior radiation therapy, n (%)	4 (50.0)	8 (44.4)	2 (22.2)	3 (42.9)	0	3 (42.9)	20 (40.0)
Prior systemic regimens for advanced or metastatic disease, n (%)	1 (12.5)	12 (66.7)	3 (33.3)	2 (28.6)	1 (100.0)	4 (57.1)	23 (46.0)
Prior systemic therapy for advanced or metastatic disease, n (%)							
Immunotherapy	0	6 (33.3)	3 (33.3)	1 (14.3)	0	1 (14.3)	11 (22.0)
Immune checkpoint inhibitor	0	3 (16.7)	2 (22.2)	1 (14.3)	0	0	6 (12.0)
Interferon	0	3 (16.7)	1 (11.1)	0	0	1 (14.3)	5 (10.0)
IL-2	0	1 (5.6)	1 (11.1)	0	0	0	2 (4.0)
Other	0	1 (5.6)	1 (11.1)	0	0	0	2 (4.0)
BRAF inhibitor	0	3 (16.7)	0	0	0	1 (14.3)	4 (8.0)
Chemotherapy	0	2 (11.1)	0	1 (14.3)	0	2 (28.6)	5 (10.0)
Other	0	4 (22.2)	1 (11.1)	2 (28.6)	1 (100.0)	1 (14.3)	9 (18.0)

*BID* twice daily, *CNS* central nervous system, *cont*’ continuous, *ECOG PS* Eastern Cooperative Oncology Group performance status, *IL-2* interleukin 2, *int’* intermittent, *LDH* lactate dehydrogenase

^a^ Epacadostat 100-mg BID and 300-mg BID dose cohorts were not re-explored in this study after protocol amendment to evaluate lower doses of epacadostat

^b^ Patient was incorrectly staged as IIIB by study site; this patient had metastases in the liver and therefore should have been reported as stage IV

### Safety and tolerability

All 50 patients received ≥1 dose of epacadostat, with a median (range) exposure of 84 (1–1352) days (Additional file 1: Table S1). Because of the protocol amendment to evaluate lower doses of epacadostat (< 100 mg BID), durations of exposure in the 100-mg BID (1 day) and 300-mg BID (median, 41 days) cohorts were shorter than in other dose cohorts. Forty-nine patients received ≥1 dose of ipilimumab (median dose/cycle, 3 mg/kg); the 1 patient treated with epacadostat 100 mg BID starting dose received only 1 dose of epacadostat and did not receive ipilimumab (Additional file 1: Table S1). The majority of patients (*n* = 28; 56.0%) received all 4 planned doses of ipilimumab. Among the 42 patients treated with epacadostat ≤50 mg BID, the median exposure to epacadostat ranged from 79 to 239 days; 27 patients (64%) received all 4 doses of ipilimumab.

Dose-limiting toxicities were reported in 11 patients, including 4 patients treated with epacadostat 300 mg BID (Additional file 1: Table S2). The most common DLT was AST and/or ALT elevation (*n* = 6), followed by colitis (*n* = 2), diarrhea (*n* = 1), pneumonitis (*n* = 1), and rash (*n* = 1). As of the data cutoff for this report, all DLTs were manageable and resolved with study drug interruption/discontinuation and treatment with corticosteroids or other standard supportive care, with the exception of 1 patient treated with epacadostat 25 mg BID who had grade 3 AST elevation that did not resolve.

Treatment-emergent AEs observed in each dose cohort are shown in Table 3a. In the overall population (*N* = 50), treatment-emergent AEs occurred in 48 patients (96%); grade 3/4 treatment-emergent AEs were reported in 33 patients (66%). The most common treatment-emergent AEs (occurring in ≥20% of all patients) were fatigue (64%), rash (52%), constipation (40%), pruritus (38%), diarrhea (34%), nausea (32%), ALT elevation (28%), decreased appetite (26%), headache (26%), AST elevation (24%), vomiting (24%), cough (22%), and arthralgia (20%). Grade 3/4 treatment-emergent AEs observed in >1 patient included ALT elevation, AST elevation (*n* = 8 each); colitis (*n* = 4); fatigue (*n* = 3); anemia, confusional state, hyperglycemia, hyponatremia, hypotension, pruritus, and urinary tract infection (*n* = 2 each). Of 42 patients treated with epacadostat doses ≤50 mg BID, 41 patients (98%) had treatment-emergent AEs; 27 patients (64%) had grade 3/4 events. The most common treatment-emergent AEs among these patients (≥20%) were fatigue (67%), rash (50%), diarrhea (36%), pruritus (36%), constipation (33%), nausea (31%), headache (29%), decreased appetite (26%), vomiting (26%), ALT elevation (21%), and cough (21%). Grade 3/4 treatment-emergent AEs occurring in >1 patient treated with epacadostat doses ≤50 mg BID were AST elevation, colitis (*n* = 4 each); ALT elevation, fatigue (*n* = 3 each); and confusional state, hyperglycemia, hyponatremia, hypotension, and urinary tract infection (*n* = 2 each).

**Table 3:** Safety Summary

Patient, n (%)	25 mg BID (*n* = 8)	50 mg BID Cont’ (*n* = 18)	50 mg BID Int’ (*n* = 9)	75 mg Total Daily (*n* = 7)	100 mg BID^a^ (*n* = 1)	300 mg BID^a^ (*n* = 7)	Total (*N* = 50)
A. Treatment-Emergent Adverse Events							
Any grade (≥10% of total patients)							
Fatigue	6 (75.0)	13 (72.2)	5 (55.6)	4 (57.1)	0	4 (57.1)	32 (64.0)
Rash^b^	4 (50.0)	10 (55.6)	1 (11.1)	6 (85.7)	0	5 (71.4)	26 (52.0)
Constipation	1 (12.5)	8 (44.4)	2 (22.2)	3 (42.9)	0	6 (85.7)	20 (40.0)
Pruritus^c^	1 (12.5)	6 (33.3)	5 (55.6)	3 (42.9)	0	4 (57.1)	19 (38.0)
Diarrhea	3 (37.5)	6 (33.3)	3 (33.3)	3 (42.9)	0	2 (28.6)	17 (34.0)
Nausea	4 (50.0)	4 (22.2)	4 (44.4)	1 (14.3)	0	3 (42.9)	16 (32.0)
ALT elevation	2 (25.0)	3 (16.7)	2 (22.2)	2 (28.6)	0	5 (71.4)	14 (28.0)
Decreased appetite	0	5 (27.8)	4 (44.4)	2 (28.6)	0	2 (28.6)	13 (26.0)
Headache	5 (62.5)	6 (33.3)	0	1 (14.3)	0	1 (14.3)	13 (26.0)
AST elevation	2 (25.0)	3 (16.7)	1 (11.1)	1 (14.3)	0	5 (71.4)	12 (24.0)
Vomiting	1 (12.5)	5 (27.8)	3 (33.3)	2 (28.6)	0	1 (14.3)	12 (24.0)
Cough	4 (50.0)	3 (16.7)	2 (22.2)	0	0	2 (28.6)	11 (22.0)
Arthralgia	3 (37.5)	1 (5.6)	3 (33.3)	1 (14.3)	0	2 (28.6)	10 (20.0)
Pyrexia	2 (25.0)	3 (16.7)	2 (22.2)	1 (14.3)	0	1 (14.3)	9 (18.0)
Abdominal pain	2 (25.0)	3 (16.7)	1 (11.1)	1 (14.3)	0	1 (14.3)	8 (16.0)
Insomnia	1 (12.5)	4 (22.2)	0	2 (28.6)	0	1 (14.3)	8 (16.0)
Chills	3 (37.5)	1 (5.6)	1 (11.1)	1 (14.3)	0	1 (14.3)	7 (14.0)
Dizziness	1 (12.5)	3 (16.7)	1 (11.1)	1 (14.3)	0	1 (14.3)	7 (14.0)
Anemia	1 (12.5)	1 (5.6)	1 (11.1)	1 (14.3)	0	2 (28.6)	6 (12.0)
Myalgia	2 (25.0)	2 (11.1)	0	0	0	2 (28.6)	6 (12.0)
Hypothyroidism	2 (25.0)	3 (16.7)	0	0	0	0	5 (10.0)
Upper respiratory tract infection	0	2 (11.1)	0	3 (42.9)	0	0	5 (10.0)
Grade 3/4 (> 1 patient total)							
ALT elevation	0	2 (11.1)	0	1 (14.3)	0	5 (71.4)	8 (16.0)
AST elevation	1 (12.5)	2 (11.1)	0	1 (14.3)	0	4 (57.1)	8 (16.0)
Colitis	2 (25.0)	1 (5.6)	1 (11.1)	0	0	0	4 (8.0)
Fatigue	0	2 (11.1)	1 (11.1)	0	0	0	3 (6.0)
Anemia	0	0	1 (11.1)	0	0	1 (14.3)	2 (4.0)
Confusional state	0	2 (11.1)	0	0	0	0	2 (4.0)
Hyperglycemia	0	1 (5.6)	0	1 (14.3)	0	0	2 (4.0)
Hypernatremia	0	1 (5.6)	0	1 (14.3)	0	0	2 (4.0)
Hypotension	0	2 (11.1)	0	0	0	0	2 (4.0)
Pruritus^c^	0	1 (5.6)	0	0	0	1 (14.3)	2 (4.0)
Urinary tract infection	1 (12.5)	0	1 (11.1)	0	0	0	2 (4.0)
B. Immune-Related Adverse Events							
Any grade (≥10% of total patients)							
Rash^b^	4 (50.0)	10 (55.6)	1 (11.1)	5 (71.4)	0	5 (71.4)	25 (50.0)
ALT elevation	2 (25.0)	3 (16.7)	2 (22.2)	2 (28.6)	0	5 (71.4)	14 (28.0)
Pruritus^c^	1 (12.5)	5 (27.8)	3 (33.3)	1 (14.3)	0	4 (57.1)	14 (28.0)
AST elevation	2 (25.0)	3 (16.7)	1 (11.1)	1 (14.3)	0	5 (71.4)	12 (24.0)
Hypothyroidism	2 (25.0)	3 (16.7)	0	0	0	0	5 (10.0)
Grade 3/4 (> 1 patient total)							
ALT elevation	0	2 (11.1)	0	1 (14.3)	0	5 (71.4)	8 (16.0)
AST elevation	1 (12.5)	2 (11.1)	0	1 (14.3)	0	4 (57.1)	8 (16.0)
Colitis	2 (25.0)	1 (5.6)	1 (11.1)	0	0	0	4 (8.0)
Pruritus^c^	0	1 (5.6)	0	0	0	1 (14.3)	2 (4.0)

*ALT* alanine aminotransferase, *AST* aspartate aminotransferase, *BID* twice daily, *cont*’ continuous, *int*’ intermittent, *MedDRA* Medical Dictionary for Regulatory Activities

^a^ Epacadostat 100-mg BID and 300-mg BID dose cohorts were not re-explored in this study after protocol amendment to evaluate lower doses of epacadostat

^b^ Rash included the following MedDRA preferred terms: rash, rash maculopapular, rash pruritic, rash generalized, and rash macular; patients were counted only once (a patient with multiple terms for rash was counted only once for the term “rash”)

^c^ Pruritus included the following MedDRA preferred terms: pruritus and pruritus generalized; patients were counted only once (a patient with multiple terms for pruritus was counted only once for the term “pruritus”)

In the overall population (*N* = 50), treatment-emergent AEs led to discontinuation of treatment in 20 patients (40%). Five of these 20 patients were initially treated with epacadostat 300 mg BID and discontinued because of ALT/AST elevations occurring either while patients were receiving the 300-mg BID dose (*n* = 3) or after the epacadostat dose was reduced to 100 mg BID (*n* = 2). Although these patients improved and recovered normal liver function after treatment with intravenous and oral corticosteroids, epacadostat doses ≥100 mg BID were not re-explored in this study. The remaining 15 patients who discontinued study treatment because of treatment-emergent AEs received epacadostat ≤50 mg BID. The only adverse event that led to discontinuation in >1 patient was colitis (*n* = 3)**.** Five patients (10%) died due to an adverse event but none were reported as treatment-related.

Immune-related AEs identified in the overall population (*N* = 50) are shown in Table 3b. Forty patients (80%) experienced irAEs of any grade, and 14 patients (28%) experienced grade 3/4 irAEs. The most common (≥10%) irAEs were rash (50%), ALT elevation (28%), pruritus (28%), AST elevation (24%), and hypothyroidism (10%). Grade 3/4 irAEs occurring in >1 patient were ALT elevation (*n* = 8), AST elevation (*n* = 8), colitis (*n* = 4), and pruritus (*n* = 2). Of 42 patients treated with epacadostat doses ≤50 mg BID, 79% experienced irAEs; 21% had grade 3/4 irAEs. The most common irAEs in these patients (≥10%) were rash (48%), pruritus (24%), ALT elevation (21%), AST elevation (17%), hypothyroidism (12%), and colitis (10%). Grade 3/4 irAEs occurring in >1 patient were AST elevation (*n* = 4), colitis (*n* = 4), and ALT elevation (*n* = 3). Immune-related AEs were generally reversible with appropriate corticosteroid treatment in addition to dose reduction or interruption of epacadostat and ipilimumab.

Overall, no significant changes in hematology parameters (platelets, hemoglobin, absolute neutrophil count, white blood cell count, and lymphocyte counts) were observed with treatment throughout the study. In addition, there was no significant change in vital signs, significant cardiac abnormalities affecting study treatment, or report of serotonin syndrome.

### Efficacy

**Fig. 1:**
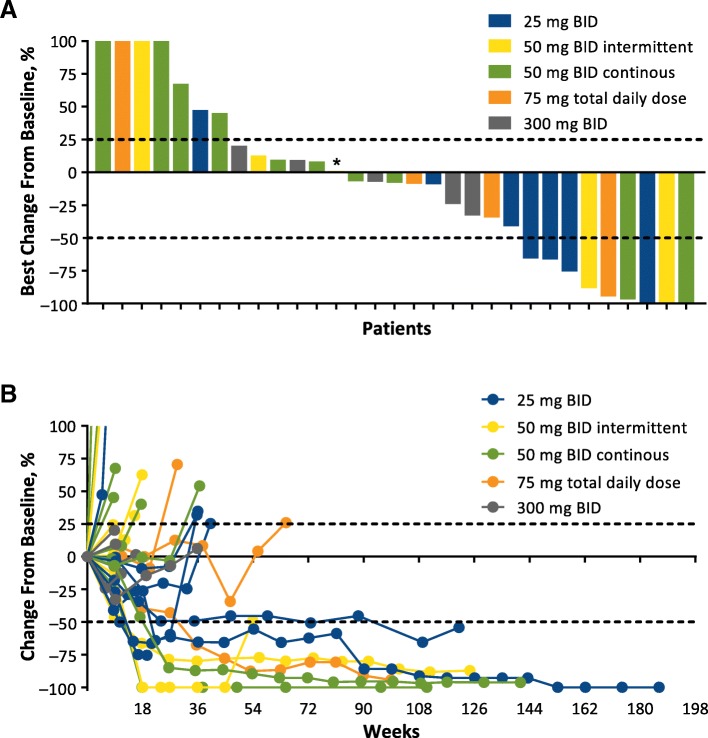
Change From Baseline in Target Lesions in Immunotherapy-Naive Patients by irRC. **a** Best percentage change from baseline in target lesions and (**b**) percentage change in target lesions over time. BID, twice daily; irRC, immune-related response criteria. * 50 mg BID intermittent; best change from baseline was 0.98%. Of 39 efficacy-evaluable immunotherapy-naive patients, data are shown for the 31 patients with postbaseline scans that included assessment of target lesions. Y axis values were shown as a maximum of 100% for readability; actual values for the first 4 bars from the left in (**a**) were 687, 259, 181, and 117%

Efficacy was evaluable in all 50 patients enrolled, including 39 immunotherapy-naive patients and 11 patients with prior immunotherapy for advanced or metastatic disease. Of 39 immunotherapy-naive patients, objective responses were observed in 10 patients (26%) by irRC (CR, 8%; PR, 18%) and 9 patients (23%) by RECIST v1.1 (CR, 8%; PR, 15%; Table 4 and Fig. 1). Median (range) DOR was 392 (148–1149+) days by irRC and 657 (114–1177+) days by RECIST v1.1. Twenty-five immunotherapy-naive patients achieved disease control by irRC (64%; 15 SD) and 19 by RECIST v1.1 (49%; 10 SD). No objective response was seen in the 11 patients who received prior immunotherapy, although 4 patients (36%) and 3 patients (27%) had SD by irRC and RECIST v1.1, respectively (Table 4).

**Table 4:** Best Objective Response by irRC and RECIST v1.1

n (%)	irRC		RECIST	
	Immunotherapy-Naive (*n* = 39)	Prior Immunotherapy (*n* = 11)	Immunotherapy-Naive (*n* = 39)	Prior Immunotherapy (*n* = 11)
Objective response rate	10 (25.6)	0	9 (23.1)	0
Complete response	3 (7.7)	0	3 (7.7)	0
Partial response	7 (17.9)	0	6 (15.4)	0
Stable disease	15 (38.5)	4 (36.4)	10 (25.6)	3 (27.3)
Disease control rate	25 (64.1)	4 (36.4)	19 (48.7)	3 (27.3)
Progressive disease	7 (17.9)	5 (45.5)	15 (38.5)	6 (54.5)
Missing	7 (17.9)	2 (18.2)	5 (12.8)	2 (18.2)

*irRC* immune-related response criteria, *RECIST* Response Evaluation Criteria in Solid Tumors

**Fig. 2:**
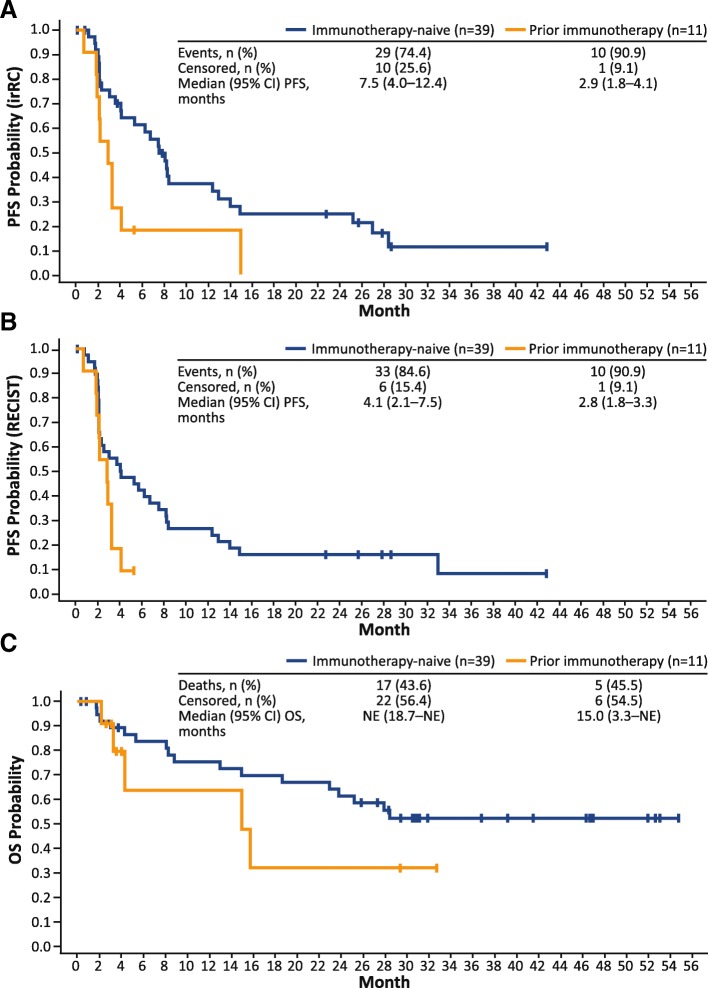
Survival Estimates by Prior Immunotherapy Status. **a** Kaplan-Meier–estimated PFS by irRC, (**b**) Kaplan-Meier–estimated PFS by RECIST, and (**c**) Kaplan-Meier–estimated OS. irRC, immune-related response criteria; NE, not evaluable; OS, overall survival; PFS, progression-free survival; RECIST, Response Evaluation Criteria in Solid Tumors

Among immunotherapy-naive patients, the median (95% CI) PFS was 7.5 (4.0–12.4) months by irRC and 4.1 (2.1–7.5) months by RECIST v1.1 (Fig. 2a and b). Median (95% CI) PFS in patients previously treated with immunotherapy was 2.9 (1.8–4.1) months by irRC and 2.8 (1.8–3.3) months by RECIST v1.1. Among all efficacy-evaluable patients, OS rates at 3, 6, 9, and 12 months were 92, 80, 73, and 73%, respectively. The median (95% CI) OS was not reached (18.7–NE months) in immunotherapy-naive patients, and was 15.0 (3.3–NE) months in patients previously treated with immunotherapy (Fig. 2c).

Efficacy assessments were limited for epacadostat 100-mg BID (*n* = 1; 1 day exposure to study drug) and 300-mg BID (*n* = 7; 41 days median exposure to study drug) dose cohorts. The 1 patient treated with epacadostat 100 mg BID discontinued treatment before the first tumor assessment. Best objective response in patients treated with epacadostat 300 mg BID was SD by irRC (*n* = 6) and RECIST v1.1 (*n* = 2).

### Pharmacokinetics

Thirty-six patients were evaluable for pharmacokinetic analyses. Patients who received epacadostat 75 mg total daily dose (50 mg am, 25 mg pm) were excluded from pharmacokinetic analyses because they were at neither the steady state of 50 mg once daily nor 50 mg BID. Steady-state pharmacokinetic observations (Cycle 1, Day 10) are summarized in Additional file 1: Table S3.

Epacadostat was absorbed rapidly after repeat oral doses of 25 mg BID, 50 mg BID, 50 mg BID intermittent, and 300 mg BID; median time of maximum observed concentration (t_max_) was approximately 2 h. Subsequently, plasma concentrations decreased in either a monoexponential or biexponential pattern; mean terminal elimination half-life (t_½_) was approximately 5 to 9 h. The observed geometric mean of exposures (maximum observed concentration [C_max_] and area under the concentration vs time curve from 0 to 12 h [AUC_0–12h_]) suggested that the epacadostat pharmacokinetic exposures increased in a dose-proportional fashion with increasing dose between 25 mg BID and 300 mg BID. In addition, the geometric mean AUC for 300 mg BID of epacadostat was 9.2 μM·h in this study, compared with 9.8 μM·h among patients with advanced malignancies treated with epacadostat monotherapy 300 mg BID in a separate study [18].

### Pharmacodynamics

Plasma kyn levels were elevated in a majority of patients at baseline (*n* = 47; median, 2200 nM) relative to previously reported levels in fasted normal healthy volunteers (median, 1499 nM) [18]. Approximately half of the baseline kyn levels are attributable to dietary metabolism of trp by liver tryptophan 2,3-dioxygenase and are not influenced by IDO1 activity [18]. Therefore, the maximal level of inhibition expected with this measure is approximately 50%. Overall, there was an average 36% reduction in the elevated baseline kyn levels when on treatment, which was consistent with the pharmacokinetic exposures achieved in this study. There was a trend toward greater reduction of kyn levels and the kyn/trp ratio with higher doses of epacadostat, suggesting a dose-dependent inhibition of IDO1 by epacadostat.

The ex vivo whole blood–based pharmacodynamic analysis based on inhibition of induced IDO1 activity confirmed a dose-dependent IDO1 inhibition at tested time points (Additional file 1: Table S4). Maximal average inhibition of 85% was achieved within the first 6 h of epacadostat 300-mg BID dosing. At doses of 25 mg BID and 50 mg BID, the average inhibition exceeded 50%. This level of IDO1 inhibition was similar to that observed in plasma when achieving maximal therapeutic effect in preclinical models [26]. In addition, these results were generally consistent with previously reported data in patients with advanced malignancies [18] and with in vitro–derived human protein-binding adjusted IC_50_ for epacadostat.

At baseline, average levels of inflammatory cytokines and protein markers (eg, C-reactive protein [CRP], interleukin [IL]-6, tumor necrosis factor-α, vascular cell adhesion molecule 1) were markedly elevated compared with healthy volunteers (data not shown). These findings were consistent with the increased inflammatory state observed in patients with advanced cancer [27–29]. A limited number of immune-related plasma cytokines and protein markers (ie, IP-10, CXCL13, CRP, IL-6) were observed to be consistently increased by <2-fold at Cycle 2 Day 1 and Cycle 4 Day 1 on study treatment compared with Cycle 1 Day 1, regardless of study treatment dose. There was no association between these elevations and response to treatment, and such changes appeared to be a pharmacodynamic effect of the combination.

## Discussion

Results of this open-label phase 1 study suggested that epacadostat at doses ≤50 mg BID was generally well tolerated when combined with ipilimumab 3 mg/kg Q3W and that this combination immunotherapy strategy demonstrated promising clinical activity in patients with unresectable or metastatic melanoma.

Although not directly comparable, the safety profile for epacadostat plus ipilimumab in this study was generally consistent with the previously reported ipilimumab monotherapy safety profile [3, 4], suggesting that epacadostat dosing of 50 mg BID or lower was associated with minimal additive AEs when combined with ipilimumab. Patients treated with epacadostat in combination with ipilimumab at doses of ≤50 mg BID in this study had grade 3/4 treatment-emergent AEs rate of 64%, whereas such AEs were observed in 56% of patients receiving ipilimumab monotherapy and 69% of patients receiving nivolumab and ipilimumab combination therapy in a separate clinical study [6]. Furthermore, 28% of patients in this study experienced grade 3/4 irAEs, which was slightly higher than the rates of irAEs previously reported with ipilimumab monotherapy (19%), [6] although it’s noteworthy that only 21% of the 42 patients treated with epacadostat doses ≤50 mg BID had grade 3/4 irAEs. The irAEs observed in this study were reversible with corticosteroids and dose reduction/interruption of study treatment. No treatment-related AEs led to death. However, it should be noted that epacadostat 300 mg BID in combination with ipilimumab 3 mg/kg was associated with unacceptable rates of grade 3/4 ALT/AST elevations, and rates of ALT and AST elevations in patients treated with epacadostat ≤50 mg BID plus ipilimumab (21 and 17%, respectively) remained higher than would be expected with ipilimumab monotherapy. Due to hepatotoxicity, epacadostat doses of 100 mg BID or higher were not explored further in this study.

The pharmacokinetic and pharmacodynamic observations of epacadostat in this melanoma study are generally consistent with previous reports in patients with advanced malignancies [18]. Epacadostat at doses of 25 mg BID, 50 mg BID, 50 mg BID intermittent, and 300 mg BID were associated with a t_max_ of approximately 2 h and a mean terminal t_1/2_ of 5 to 9 h. Additionally, the level of IDO1 enzyme inhibition achieved with epacadostat doses ≥25 mg BID in this study was similar to levels associated with maximal therapeutic effect in preclinical models.

Efficacy findings in this study demonstrate that immunotherapy-naive patients with unresectable or metastatic melanoma may benefit from treatment with epacadostat plus ipilimumab based on the ORR (26% per irRC; 23% per RECIST v1.1), CR (8% per irRC and RECIST v1.1), disease control rate (64% per irRC; 49% per RECIST v1.1), PFS (median [95% CI]: 4.1 [2.1–7.5] months by RECIST; 7.5 [4.0–12.4] months by irRC), and OS (median [95% CI]: not reached [18.7–NE] months). Although no objective responses were observed among patients previously treated with immunotherapy, SD was achieved in 36% of patients by irRC and 27% by RECIST v1.1.

Together, these findings suggest that epacadostat in combination with ipilimumab is active in patients with metastatic melanoma, but additional evidence is required to further assess the potential therapeutic benefits of this combination. At the time of this publication, results from the pivotal phase 3 ECHO-301/KEYNOTE-252 trial (NCT02752074) demonstrated that the combination of epacadostat 100 mg BID and pembrolizumab (anti–PD-1 inhibitor) 200 mg Q3W failed to reach the primary endpoint of improved PFS compared with pembrolizumab monotherapy in patients with unresectable/metastatic melanoma [30]. Given these findings, the role of IDO1 inhibitors in general, and epacadostat in particular, in melanoma and other advanced malignancies remains unclear at this time. Future results from ongoing randomized clinical studies may provide insights into whether a role exists for epacadostat-based combination strategies. Nonetheless, the response rate observed in the current study may warrant continued investigation of epacadostat (or other IDO1 inhibitors) in combination with anti–CTLA-4 antibody therapy.

## Conclusion

This was the first study to evaluate an IDO1 inhibitor in combination with ipilimumab in the clinical setting. Although preliminary, the results from this study suggest that epacadostat ≤50 mg BID in combination with ipilimumab 3 mg/kg has an acceptable safety profile, and that this combination has the potential to enhance clinical activity in patients with unresectable or metastatic melanoma, particularly in those who have not been previously treated with immunotherapy.

## Additional file

Additional file 1: **Figure S1.** Study Design. **Table S1.** Epacadostat and Ipilimumab Treatment Exposure. **Table S2.** Dose-Limiting Toxicities. **Table S3.** Epacadostat Steady-State Pharmacokinetic Parameters (Cycle 1, Day 10). **Table S4.** Whole Blood Kynurenine Pharmacodynamics Analysis. (PDF 214 kb)

## Abbreviations

AE: Adverse event
ALT: Alanine aminotransferase
AST: Aspartate aminotransferase
AUC_0–12h_: Area under the concentration vs time curve from 0 to 12 h
BID: Twice daily
C_max_: Maximum observed concentration
CNS: Central nervous system
cont’: Continuous
CR: Complete response
CRP: C-reactive protein
CTLA-4: Cytotoxic T-lymphocyte-associated protein 4
DLT: Dose-limiting toxicity
DOR: Duration of response
ECOG PS: Eastern Cooperative Oncology Group performance status
FDA: United States Food and Drug Administration
IDO1: Indoleamine 2,3-dioxygenase 1
IL: Interleukin
int’: Intermittent
irAE: Immune-related adverse event
irRC: Immune-related response criteria
kyn: Kynurenine
LC-MS/MS: Liquid chromatography with tandem mass spectrometry
LDH: Lactate dehydrogenase
MedDRA: Medical Dictionary for Regulatory Activities
NE: Not evaluable
ORR: Objective response rate
OS: Overall survival
PD: Progressive disease
PD-1: Programmed cell death protein 1
PD-L1: PD-ligand 1
PFS: Progression-free survival
PR: Partial response
Q3W: Every 3 weeks
RECIST: Response Evaluation Criteria in Solid Tumors
SD: Stable disease
t_½_: Terminal elimination half-life
t_max_: Time of maximum observed concentration
TME: Tumor microenvironment
TRAE: Treatment-related AE
trp: Tryptophan
VEGF: Vascular endothelial growth factor
