# Supplementary material for: Phase 1/2 study of epacadostat in combination with ipilimumab in patients with unresectable or metastatic melanoma
Source: J Immunother Cancer. 2019 Mar 20;7:80. doi: 10.1186/s40425-019-0562-8 (PMC6425606; doi:10.1186/s40425-019-0562-8)
Supplement: Supplementary file 1 — Figure S1. Study Design. Table S1. Epacadostat and Ipilimumab Treatment Exposure. Table S2. Dose-Limiting Toxicities. Table S3. Epacadostat Steady-State Pharmacokinetic Parameters (Cycle 1, Day 10). Table S4. Whole Blood Kynurenine Pharmacodynamics Analysis. (PDF 214 kb) [file 40425_2019_562_MOESM1_ESM.pdf]

Phase 1/2 Study of Epacadostat in Combination With Ipilimumab in Patients With Unresectable or Metastatic Melanoma

Geoffrey T. Gibney, Omid Hamid, Jose Lutzky, Anthony J. Olszanski, Tara C. Mitchell, Thomas F. Gajewski, Bartosz Chmielowski, Brent A. Hanks, Yufan Zhao, Robert C. Newton, Janet Maleski, Lance Leopold, Jeffrey S. Weber

SUPPLEMENTARY MATERIALS

Supplementary Figure S1. Study Design………………………………………………………………………page 2

Supplementary Table S1. Epacadostat and Ipilimumab Treatment Exposure…………………page 3

Supplementary Table S2. Dose-Limiting Toxicities………………………………………………………page 4

Supplementary Table S3. Epacadostat Steady-State Pharmacokinetic Parameters

(Cycle 1, Day 10)……………………………………………………………………………………………………..page 5

Supplementary Table S4. Whole Blood Kynurenine Pharmacodynamics Analysis…………..page 6

Supplementary Figure S1. Study Design

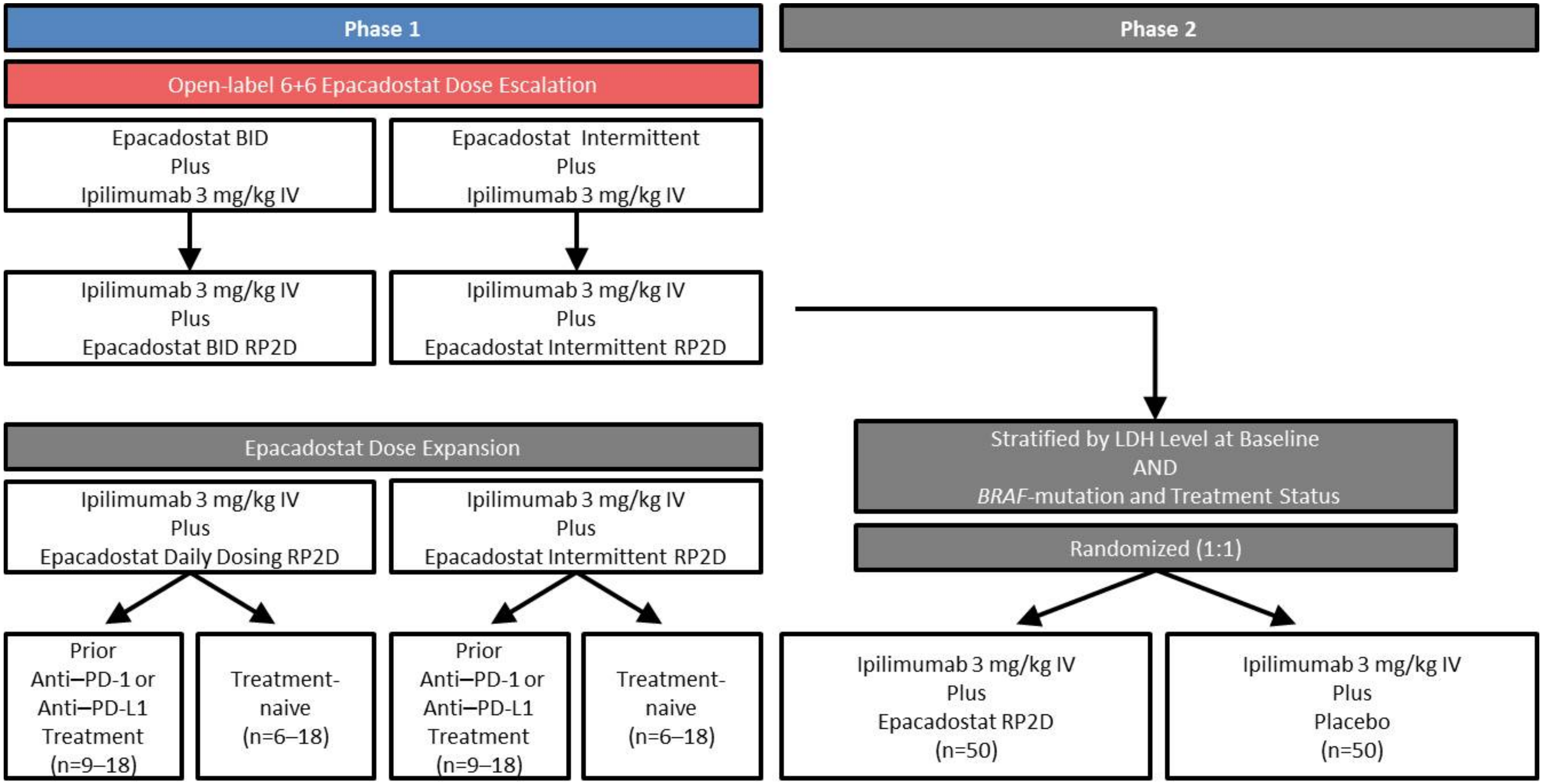

BID, twice daily; IV, intravenous; LDH, lactate dehydrogenase; PD-1, programmed cell death protein 1; PD-L1, programmed cell death ligand 1; RP2D, recommended phase 2 dose.
Note: Phase 1 expansion cohorts and phase 2 were not conducted.

Supplementary Table S1. Epacadostat and Ipilimumab Treatment Exposure

	25 mg BID (n=8)	50 mg BID Cont’ (n=18)	50 mg BID Int’ (n=9)	75 mg Total Daily (n=7)	100 mg BID* (n=1)	300 mg BID* (n=7)	Total (N=50)
Median (range) days of exposure to epacadostat	239 (9–1352)	79 (10–1019)	109 (7–941)	211 (24–739)	1 (1–1)	41 (30–75)	84 (1–1352)
Median (range) average daily dose of epacadostat, mg	48 (39–50)	100 (50–100)	71 (54–100)	75 (27–75)	100 (100–100)	332 (296–600)	76 (27–600)
Patients by total ipilimumab dose received, n							
0 dose	0	0	0	0	1[†]	0	1[†]
1 dose	1	1	1	0	0	0	3
2 doses	1	3	1	1	0	5	11
3 doses	1	3	1	1	0	1	7
4 doses	5	11	6	5	0	1	28

BID, twice daily; cont’, continuous; int’, intermittent.

* Epacadostat 100-mg BID and 300-mg BID dose cohorts were not re-explored in this study after protocol amendment to evaluate lower doses of epacadostat.

[†] The 1 patient in the 100-mg BID dose cohort discontinued study after 1 day of treatment with epacadostat and did not receive ipilimumab.

Supplementary Table S2. Dose-Limiting Toxicities

Epacadostat Dose Cohort	Number of Patients With DLT/ Number of Evaluable Patients	DLT Description
25 mg BID	1/8	Grade 3 AST elevation (n=1)*
50 mg BID cont’	4/18	Grade 3 ALT/AST elevation (n=1)<br>Grade 3 colitis (n=1)<br>Grade 3 diarrhea (n=1)<br>Grade 3 pneumonitis (n=1)
50 mg BID int’	1/9	Grade 3 colitis (n=1)
75 mg total daily	1/7	Grade 3 rash (n=1)
100 mg BID†	0/1	–
300 mg BID†	4/7	Grade 4 ALT/AST elevation (n=1)<br>Grade 3 ALT elevation/grade 2 AST elevation (n=1)<br>Grade 3 ALT elevation (n=2)

ALT, alanine aminotransferase; AST, aspartate aminotransferase; BID, twice daily; cont’, continuous; DLT, dose-limiting toxicity; int’, intermittent.

* Patient experienced with progression of prior extensive liver metastases.

† Epacadostat 100-mg BID and 300-mg BID dose cohorts were not re-explored in this study after protocol amendment to evaluate lower doses of epacadostat.

Supplementary Table S3. Epacadostat Steady-State Pharmacokinetic Parameters (Cycle 1, Day 10)

Parameter	25 mg BID (n=10)*	50 mg BID Cont' (n=13)	50 mg BID Int' (n=7)	300 mg BID* (n=6)
C_{max}, μM	0.3±0.1 (0.3)	0.5±0.2 (0.4)	0.6±0.1 (0.5)	2.6±0.7 (2.6)
t_{max}, h	2.0 (1.0–4.0)	2.0 (1.0–4.0)	2.0 (1.0–2.2)	2.0 (1.0–2.0)
C_{min}, μM	0.03±0.03 (NC)	0.06±0.05 (NC)	0.06±0.04 (NC)	0.3±0.2 (0.2)
$AUC_{0–12h}$, μM·h	1.1±0.7 (0.9)	1.9±0.8 (1.8)	2.2±0.7 (2.1)	9.5±3.0 (9.2)
$t_{½}$, h	9.1±6.1 (7.4)	7.1±5.5 (5.9)	5.1±2.9 (4.5)	6.2±3.5 (5.4)
CL/F, L/h	68.9±32.1 (61.3)	69.7±29.8 (64.2)	57.6±21.7 (54.4)	77.6±22.2 (74.8)
V_z/F, L/h	891±840 (592)	722±547 (568)	400±229 (342)	709±512 (579)

Values are expressed as mean ± SD (geometric mean) except t_{max}, which is expressed as median (range).
AUC_{0-12h}, area under the concentration time curve from time 0–12 hours after dosing; C_{max}, maximum plasma concentration; C_{min}, minimum plasma concentration; CL/F, oral dose clearance; n, number of patients with AUC calculable; NC, not calculated; t_{max}, time to maximum plasma concentration; $t_{½}$, elimination half-life; V_z/F, volume of distribution.
* Two patients from the 50-mg BID cohort received epacadostat 25 mg BID at the time of the pharmacokinetic sample blood draw at the steady-state visit.

Supplementary Table S4. Whole Blood Kynurenine Pharmacodynamic Analysis

Dose	Patients, n	Inhibition Maximal,* %	Average Inhibition (0–6 h),* %	Inhibition Trough,* %
25 mg BID	4	60±15 (40–77)	70±9 (31–79)	48±29 (8–76)
50 mg BID cont’	14	87±12 (58–100)	70±20 (29–99)	46±38 (0–98)
50 mg BID int’	1	86	59	14
75 mg total daily	5	71±36 (7–90)	46±24 (7–69)	6±10 (0–22)
300 mg BID	3	98±2 (96–100)	85±17 (65–97)	67±23 (40–81)

BID, twice daily.

* All values are mean ± SD (range).
